# Recycled Mineral Raw Materials from Quarry Waste Using Hydrocyclones

**DOI:** 10.3390/ma12132047

**Published:** 2019-06-26

**Authors:** Menéndez-Aguado L.D., Marina Sánchez M., Rodríguez M.A., Coello Velázquez A.L., Menéndez-Aguado J.M.

**Affiliations:** 1 Escuela Politécnica de Mieres, Universidad de Oviedo, 33600 Oviedo, Spain; 2 Idonial Centro Tecnológico, Parque Tecnológico de Asturias, 33428 Llanera, Spain; 3 CETAM, Universidad de Moa Dr. Antonio Núñez Jiménez, Bahía de Moa 83300, Cuba

**Keywords:** waste, aggregates, fines processing

## Abstract

Mining activities in general, and quarrying processes in particular, generate huge amounts of tailings with a considerable presence of fine particles and with a variable composition of minerals, which could limit the direct application of those wastes. Under the paradigm of a circular economy, more effort has to be made to find adequate applications for those secondary raw materials. In this study, a process was proposed and tests were performed to valorise fine particle product as a raw material for the building and construction industry. Samples were taken from wastes in several aggregate production plants, being characterized and processed to remove the clayey components to obtain the cleanest quartz fraction. Then, different characterization and validation tests were carried out to analyse the application of these products as raw materials in the building and construction industry (cement and ceramics). Results showed that with no complex technologies, the tailings can be considered as a mineral raw material in different applications.

## 1. Introduction

The decrease in the grade of ore in the mineral deposits, with lower liberation sizes, and the growing demand for mineral products results in increasing tonnage of processed ore in the mineral processing plants at a lower grinding size [1,2]. In most of the unit operations, the efficiency of recovering such fine particles is very low [3]. The quarrying process creates a great amount of tailings with considerable presence of particles under 100 µm and of a variable mineral composition, along with other components, such as iron oxides or clayey materials, which could limit the application of those wastes. Under the paradigm of a circular economy, several researchers have emphasised the need for rethinking mining wastes and tailings as secondary raw materials [2,4,5].

Following this concept, some authors reported the use of mine tailings on mortars [6,7,8,9,10,11], ceramics [12], and other applications [13]. In this study, a process was proposed that can valorise a product as a raw material to the building and construction industry [14,15]. Samples were taken from wastes in several quartz aggregate production plants in Asturias (Spain); after mineral sample characterization, they were processed to remove the clayey component and to obtain the cleanest quartz fraction. The designed process includes unitary operations requiring low capital and operating costs, as is the case of hydrocyclone classification. Subsequently, the application of the products in the cement and ceramics industry was investigated. 

## 2. Materials and Methods

### 2.1. Characterization Tests

To perform the mineralogical assays, a Bruker XRD model D8 Advance (Billerica, MA, USA) was used. XRF assays were made in a Bruker XRF S-4 Pioneer Advance Billerica, MA, USA), with sample preparation in a Claisse perler, model M-4 (Malvern, Worcestershire, UK). Trace element detection was carried out using inductively coupled plasma atomic emission spectroscopy (ICP-OES), in a Varian Vista-PRO.

To perform specific gravity measures, a Micromeritics He was used. Particle size distribution tests were performed in a laser diffraction device, a Beckman Coulter LS 13 320 (Brea, CA, USA).

### 2.2. Separation Tests

Hydrocyclones are classifying devices that can perform an efficient particle size separation to remove clayey particles. Furthermore, hydrocyclones are commonly used in mineral processing plants when there is a need of particle classification under 200 microns. In this study, the hydrocyclone was run in closed circuit using a Mozley C124 test rig that incorporates a bypass line to control the flow-rate/pressure to the hydrocyclone. The vortex finder and spigot sizes were 14.3 and 6.4 mm, respectively.

The hydrocyclone feed sample was pulped to about 20% solids, measured using a Marcy gauge. The hydrocyclone operating pressure was adjusted to 7 kPa to obtain a rightly shaped underflow. Then, samples of feed, underflow, and overflow slurry were collected.

Batch tests were conducted to examine the required number of cleaning stages (treatments) to get the underflow product (coarser particles) as clean as possible of clay contents, thereby increasing the quartz content. The cleaning stages means that the underflow product of a treatment is afterwards used as feed for the following treatment or cleaning stage.

### 2.3. Application Tests

The use of the recycled product as a secondary mineral raw material in mortars, white cement, and ceramics was studied.

To study the application of the final product in mortars, a reference was established as follows: a series of cast cubes (5 × 5 × 5 cm) were prepared with 75% normalized sand from the Eduardo Torroja Institute (Madrid, Spain) and 25% Portland cement CEM II/B-V 32.5R (Cementos Tudela Veguin, Asturias, Spain), the cement/water ratio being 1:0.5. Once we had prepared the mortar, it rested in the mold for 1 day and afterwards it was introduced in a wet chamber for 28 days. Finally, various groups of three cast cubes were tested in a 250 ton press with rapid breakage. Compression strength tests were carried according to the norm UNE-EN 196-1:2018 (www.une.org).

Once the reference was established, the process was repeated, replacing the sand to different degrees with the recycled product. Table 1 lists the weights used in each case.

To test the ability of application in white cement production, in which a major importance parameter is the brightness index, a colorimeter HAC model DR/890 was used to obtain the whiteness index, according to the norm EN ISO 787-16:1995 (www.une.org).

Finally, the use of the recycled product in ceramics was validated through characterization of plastic properties. In this case, the application of the clean product as a raw material in ceramics was studied, with the economy mainly conditioned by transportation costs. Table 2 shows the specification of quartz sand to be used in some ceramic materials.

It can be envisaged that, in this case, the main problems will come from the Al_2_O_3_ and TiO_2_ contents. The alumina content can be reduced because it is associated with clayey components, but this cannot be said in the case of titanium oxide. This oxide can be undesirable because it enhances the color of other oxides in the kiln (Fe, Cr, Mn, Co, Ni, Cu); therefore, an additional cleaning processes (froth flotation, leaching) will probably be necessary.

To carry out the study in ceramic mixtures, a basis clay material was selected with the composition shown in Table 3. Mixtures at different compositions were prepared to study the liquid limit, plastic limit, and plasticity index, following the norm ISO 17892-12:2018 (https://www.iso.org/standard/72017.html) Additionally, the behaviour under different thermal treatments at 300 °C, 600 °C, and 900 °C was studied.

Dimensional variation with temperature was also studied on cylinder probes with different compositions, up to 30%. Samples in the form of cast cylinders (37.7 mm diam. and 22.1 mm long) were prepared, as summarized in Table 4.

Special care was taken in the preparation procedure to avoid air bubble formation within the mass. The probes were dried at 70 °C for 18 h, then weighed and measured. Then, the cylinders were dried again at 300 °C for 18 h, and afterwards, again weighed and measured. This operation was repeated again for the treatments at 600 °C and 900 °C.

## 3. Results and Discussion

### 3.1. Material Characterization

**Figure 1:**
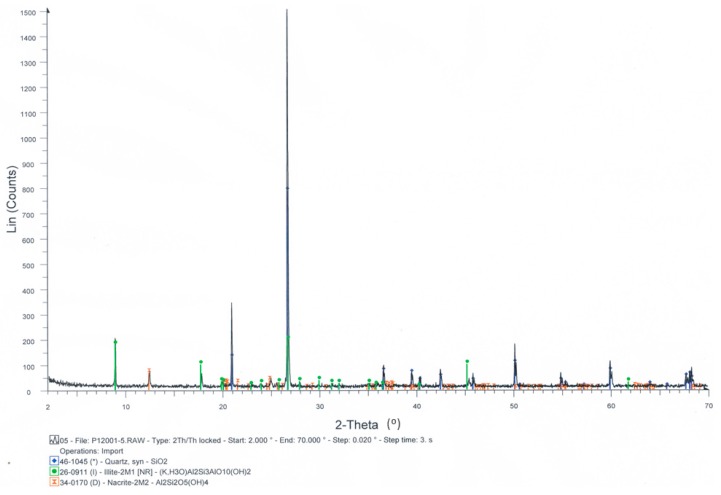
Mineralogical analysis result.

The mineralogical analysis results are shown in Figure 1, which demonstrates the presence of quartz and the presence of clay constituents such as illite and nacrite. Both complex silicates were the main problem for the direct reuse of this waste and needed to be removed in an earlier treatment.

**Figure 2:**
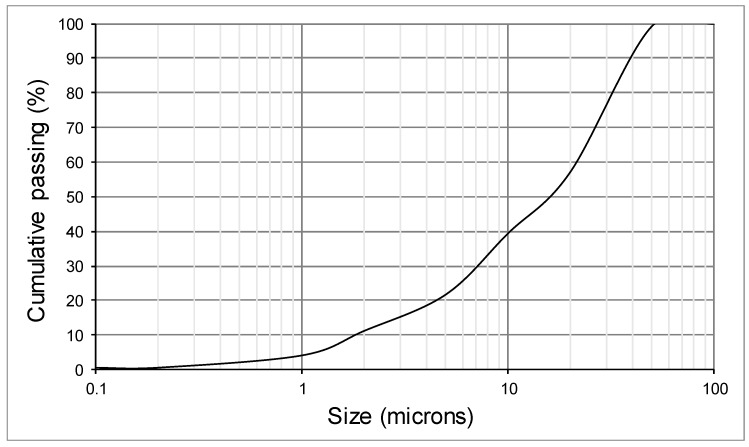
Particle size distribution.

In Figure 2, the size distribution graph obtained in the test is shown, revealing that the top size in the feed was 50 microns.

Due to the fineness of the sample, XRF analysis could be performed without further comminution, and results for major components are shown in Table 5.

To carry out the trace elements detection, ICP-OES was performed; the sample was digested with aqua regia and microwave action. Results are shown in Table 6.

To clear up any possible doubt about the environmental aspects of this secondary raw material, leaching tests were carried out to evaluate the toxicity risk; sub-samples of 10 g were taken and introduced in 50 mL water during 12 h. The liquid obtained was tested with the spectrometer ICP-OES, and the results (Table 7) showed that toxicity levels were very low.

The aforementioned presence of clay components (illite and nacrite), which are the main problems for the direct reuse of this waste, suggested the requirement for prior treatment.

### 3.2. Separation Tests

As can be seen in Table 8, the quartz content increased from 89% to 98% with four hydrocyclone treatments under the abovementioned operating conditions.

**Figure 3:**
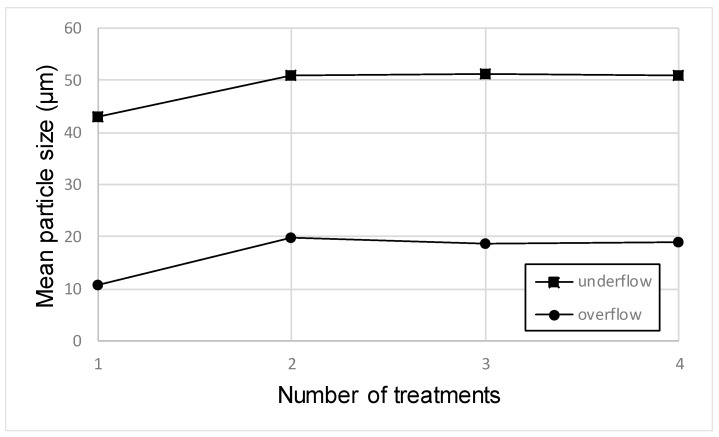
Variation of mean particle size with treatments.

Mean grain size obtained in the underflow and overflow products are shown in Figure 3. The increasing particle size with the treatments in the underflow products can be explained as follows: in each treatment, a certain amount of fine product was removed in the overflow, so the re-treatment of the underflow product meant a slightly coarser feed, which led (with the same conditions) to a slightly coarser cut size. This effect was attenuated with the number of treatments because in each step, the finer fraction was removed from the underflow product.

Results after four cleaning stages show that quartz was concentrated in the underflow product (coarser particles), while clays were concentrated in the finer fraction, reducing the Al_2_O_3_ and Fe_2_O_3_ levels in the underflow product with each treatment. Once the treatment procedure was defined, enough recycled product had been prepared under the same separation conditions to carry out the mortar tests. Due to the limited capacity of the hydrocyclon test bench (10 kg), the necessary amount of material was obtained in several batches. In all cases, feed material was adequately homogenized and sampled before batch separation. The final product obtained was also homogenized and sampled to ensure sample representativeness.

### 3.3. Application Tests

#### 3.3.1. Recycled Product in Mortars

**Figure 4:**
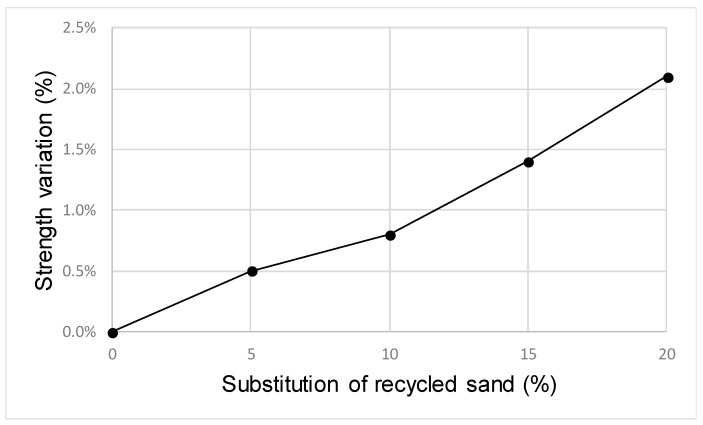
Variation in compression strength due to product addition.

Table 9 and Figure 4 show the results obtained in the compression strength tests developed in each case. The reference value was considered to be 35 MPa, without sand substitution. It must be noticed that in all cases, the difference in the strength values were lower than 5%.

#### 3.3.2. Recycled Product in White Cement Production

**Figure 5:**
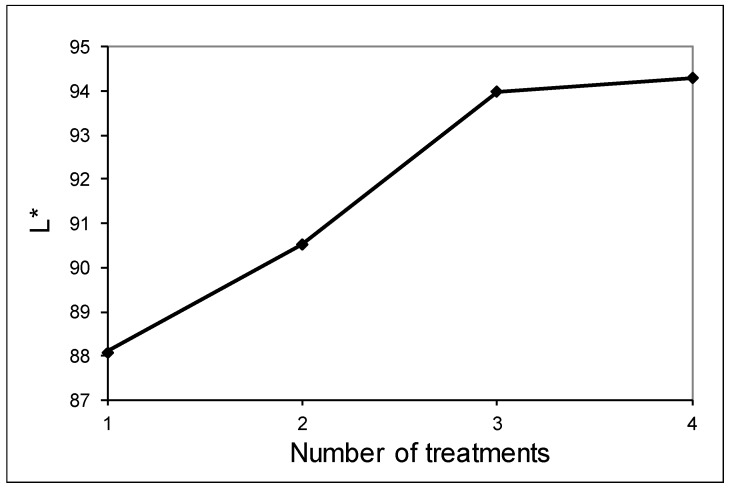
Variation of whiteness index L* with number of cleaning stages.

Initially, the underflow product from the first hydrocyclone treatment was used; its chemical composition is shown in Table 8. Due to the high SiO_2_ content (96.51%), the objective was to study the effect of replacing the quartz contribution of raw materials with this recycled product. As a result, it was found that this product was suitable for white clinker production in terms of compressive strength: strength values registered only a slight decrease and the setting times were only slightly longer. However, the whiteness level reached was not enough. The L* index of the clean product reached only 88, whereas the necessary L* additive index in the cement production process is at least 94. After obtaining this result, further studies where conduction to establish whether more hydrocyclone treatments could improve the L* index. In Figure 5, the variation of L* index is shown with the number of treatments, reaching the value 94 in the third treatment, with no sensible improvement in the fourth one.

#### 3.3.3. Recycled Product in Ceramics

**Figure 6:**
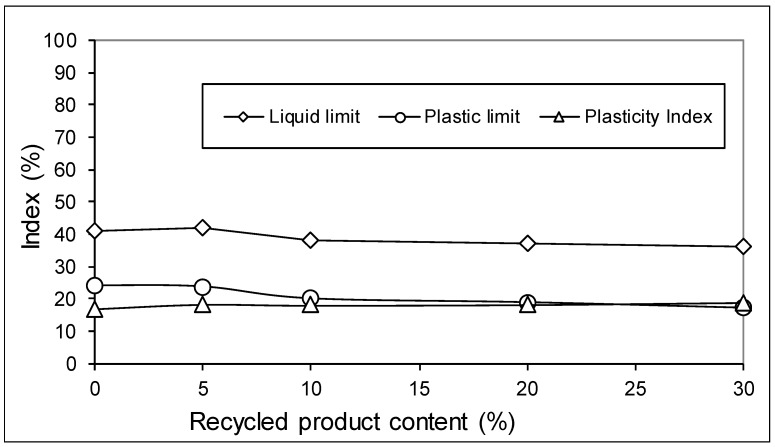
Variation of plasticity parameters.

Figure 6 shows the results obtained in the plasticity index, from which it can be concluded that the mixtures could contain up to 30% recycled product without great variation in the plasticity index.

**Figure 7:**
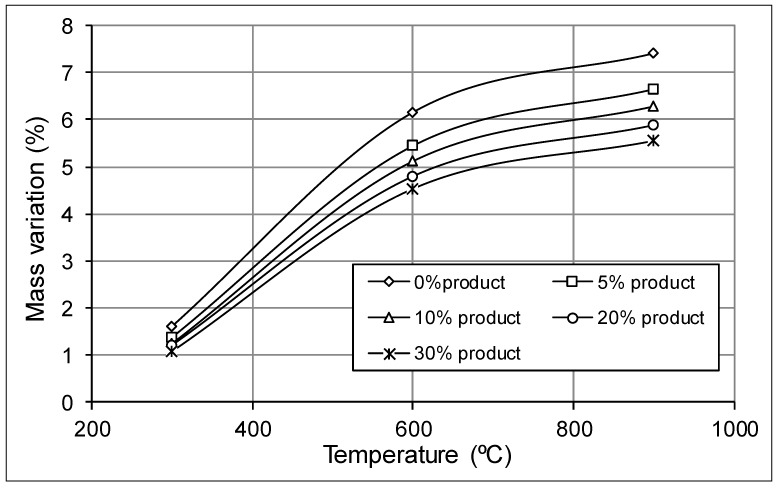
Mass variation with temperature at different recycled product content.

**Figure 8:**
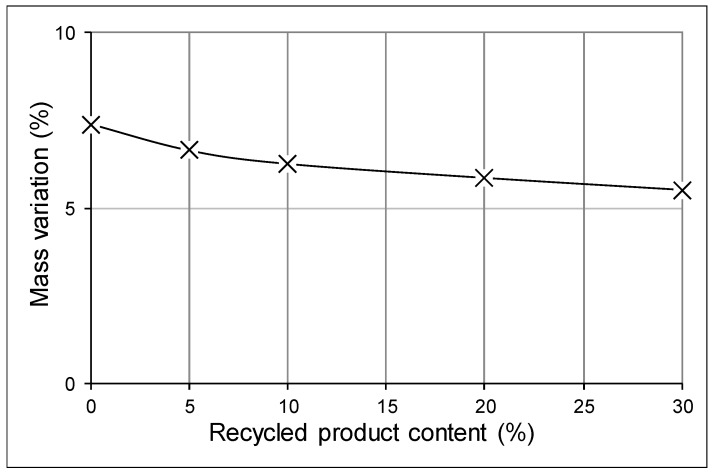
Mass variation at 900 °C.

A similar mass variation was obtained in each composition, with the higher relative variation obtained at 300 °C, probably due to the evaporation of water crystallization in the clayey minerals. The mass loss at 900° was probably due to carbide decomposition and organic matter being removed from the clays. All of these changes are shown in Figure 7 and Figure 8.

**Figure 9:**
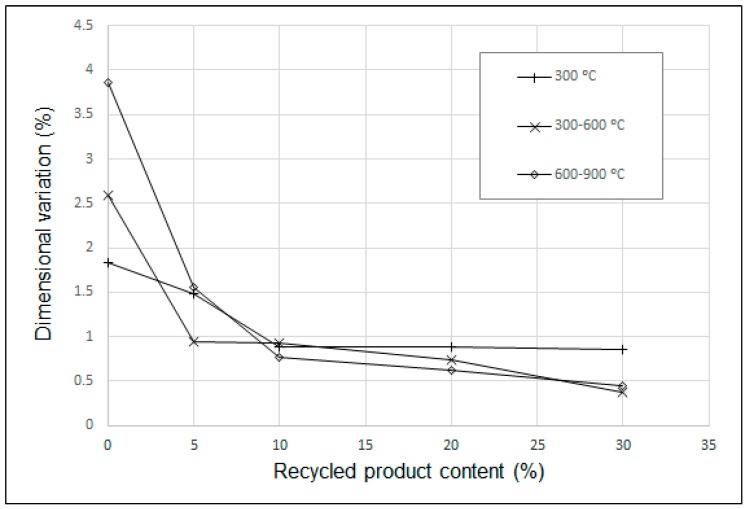
Dimensional variation at 300 °C, 300–600 °C, and 600–900 °C.

Regarding dimensional variation, results observed in each temperature range are depicted in Figure 9. It must be clarified that in the temperature ranges 0–300 °C and 600–900 °C, shrinkage was observed, while in the interval 300–600 °C, an expansion was recorded.

As observed in the curves represented in Figure 9, within the 0–10% range of sand substitution by recycled sand, more differences in the properties were measured; this effect of physical properties variations within this range was reported by Almeida et al. [10]. It must be noted here that the addition must be performed carefully to ensure the properties in the mixture; a 10% sand addition reduced the dimensional variation significantly without the appearance of cracks after thermal treatment, but this was not the case when the addition was raised until 30%.

## 4. Conclusions

- Treatment of quarry tailings with hydrocyclones is an effective methodology to turn a waste into a by-product. The underflow product can be considered a cleaned quartz fraction, with a wide range of applications under circular economy principles. In the case of some specific applications, the recycled product could not meet the necessary standard of purity due to the concentration of titanium oxides in the underflow product. In that case, further processing steps could be performed, as is the case with froth flotation.
- Use in mortars and concrete: The recycled product can replace up to 20% of the normalized sand with a compression strength reduction below 3%. Furthermore, in all cases, the recycled product substitution had no noticeable impact on the mechanical properties or the setting time in the cement.
- Use in grey clinker production: In terms of mechanical properties, the recycled product with only one cleaning stage could be used directly in grey clinker production.
- Use in white cement production: With 3–4 cleaning stages, the recycled product reached the necessary whiteness level to be a raw material for white cement production.
- Use in ceramics: The addition of up to 10% of recycled product did not have a relevant influence in the plasticity properties of the ceramic paste relative to the basis clay selected; in this case, an effect of decreasing the dimensional variation during the process within the kiln was reported, without the appearance of cracks when the addition level was set to around 10%.

## Figures and Tables

**Table 1:** Mortars prepared replacing sand by cleaned product.

Sand Substitution (%)	Normalized Sand (g)	Cement (g)	Water (g)	Cleaned Product (g)
0	700	233	116.5	0
5	665	233	116.5	35
10	630	233	116.5	70
15	595	233	116.5	105
20	560	233	116.5	140

**Table 2:** Quartz sand specifications.

Material	Fe_2_O_3_ (%)	TiO_2_ (%)	Al_2_O_3_ (%)	K_2_O (%)	CaO (%)	D_90_ (µm)
Enamel	<0.06	<0.06	<0.7	<0.2	<0.2	150
Ingobbio	<0.06	<0.06	<0.7	<0.2	<0.2	63–125
Ceramic pastes (porous/gres/porcelain)	<0.15	<0.1	3–7	2–5	<0.3	100 µm–6 mm *

* Depending on the preparation process.

**Table 3:** Chemical composition in the basis clay material.

Al_2_O_3_	SiO_2_	Fe_2_O_3_	TiO_2_	CaO	MgO	Na_2_O	K_2_O	P2O5	LOI
19.05	64.71	3.89	0.84	0.15	0.61	0.18	4.03	0.13	6.42

**Table 4:** Samples prepared to the thermal study.

Cast Cylinder	Clay (g)	Recycled Product (g)	Water (g)
1	80	0	30
2	100	0	30
3	95	0	30
4	90	0	32
5	70	0	33
6	95	5	32
7	90	10	31
8	80	20	30
9	70	30	30

**Table 5:** Major components in the sample (%).

Al_2_O_3_	SiO_2_	Fe_2_O_3_	TiO_2_	CaO	MgO	Na_2_O	K_2_O	P_2_O_5_	LOI
6.49	89.42	0.73	0.67	<0.1	<0.1	0.03	1.10	<0.1	1.54

**Table 6:** ICP-OES trace elements results (mg/kg).

As	Ba	Sr	Sb	Co	Cr	Cu	Cd	Hg	Pb	Zn	Zr	Ni	Mn	Sn
18	142	15	<10	14	31	22	<10	<10	<10	<10	85	<10	17	<10

**Table 7:** Leach test results (mg/kg).

As	Ba	Sr	Sb	Co	Cr	Cu	Cd	Hg	Pb	Zn	Zr	Ni	Mn	Sn	Al	Fe	K
<5	<5	<5	<5	<5	<5	<5	<5	<5	<5	<5	<5	<5	<5	<5	17	6	27

**Table 8:** Underflow fraction results after 3 treatments.

Product	Al_2_O_3_	SiO_2_	Fe_2_O_3_	TiO_2_	CaO	MgO	K_2_O	P_2_O_5_
Feed	6.49	89.42	0.73	0.67	<0.1	<0.1	1.10	<0.1
Underflow, treatment 1	2.19	96.51	0.11	0.48	<0.1	<0.1	0.31	<0.1
Underflow, treatment 2	1.16	96.92	0.07	0.45	<0.1	<0.1	0.15	<0.1
Underflow, treatment 3	0.92	98.05	0.05	0.42	<0.1	<0.1	0.1	<0.1
Underflow, treatment 4	0.89	98.12	0.04	0.41	<0.1	<0.1	<0.1	<0.1

**Table 9:** Compression tests results.

	Substitution of Recycled Sand (%)				
	0	5	10	15	20
**Compression tests (Mpa)**	35.4	35.1	34.9	34.7	34.6
	35.2	35.2	35.1	34.8	34.5
	35.3	35.1	35.1	34.9	34.7
**Average (Mpa)**	35.3	35.1	35.0	34.8	34.6
**Variation (%)**	–	0.5%	0.8%	1.4%	2.0%
